# Relationship between Repeated Sprint Ability, Countermovement Jump and Thermography in Elite Football Players

**DOI:** 10.3390/s23020631

**Published:** 2023-01-05

**Authors:** Carlos Majano, Jorge García-Unanue, Antonio Hernandez-Martin, Javier Sánchez-Sánchez, Leonor Gallardo, Jose Luis Felipe

**Affiliations:** 1 IGOID Research Group, Physical Activity and Sport Sciences Department, University of Castilla-La Mancha, 45071 Toledo, Spain; 2 School of Sport Sciences, Universidad Europea de Madrid, 28670 Villaviciosa de Odón, Spain

**Keywords:** asymmetries, fatigue, temperature

## Abstract

Football is a very demanding sport which requires players to exert maximum effort, producing fatigue and eventually injuries. Thermography can be used to detect fatigue and prevent its consequences through thermal asymmetries in the bilateral body areas; however, its adequacy for elite footballers has not been widely studied. Therefore, the objective of the present investigation was to determine the suitability of thermography to detect fatigue in male football players. For this reason, twenty participants were gathered into a pair of subgroups (low [<0.2 °C] vs. high thermal asymmetry [≥0.2 °C]) based on a thermography session of the lower limbs (thighs, calves, and hamstrings). After the thermography session, players performed CMJs before and after an RSA test (6 × 30 m/20″). A mixed two-way analysis of variance and Bonferroni post hoc pairwise comparisons were undertaken to analyse the results. No significant differences (*p* > 0.05) were found in any of the RSA test variables between low and high thermal asymmetry groups for thighs and calves. On the other hand, the low thermal asymmetry hamstring group reported a smaller percentage difference in sprints for the first sprint (%Diff) and a larger percentage difference in sprints two and three with respect to the best sprint (%Best). For CMJs, the low thermal asymmetry hamstring group reported significantly higher values post-RSA test, indicating better performance. Accordingly, thermography can provide information about performance in CMJ and RSA tests through hamstring asymmetries over 0.2 °C. Meanwhile, larger asymmetries than 0.2 °C in calves and thighs do not seem to be related to performance in these tests; therefore, coaches should consider if it is optimal to align players with high hamstring asymmetries.

## 1. Introduction

Injuries and performance are important factors in football, which is a sport characterized by continuous changes in activity, alternating high-intensity actions with short resting periods [1]. In addition, the game demands tackles, shots, jumps, accelerations, decelerations, changes of direction and dribbles, making the sport highly physiologically demanding [2]. This is why muscle injuries of the lower extremities occur frequently in elite and amateur football players during matches [3]; however, only 5% of these injuries occur as a consequence of faults and contacts, which means that it may be possible to prevent these injuries to some extent [4]. A congested calendar of matches and training can induce fatigue, understood as several alterations in the central nervous system that produce an inability to maintain the required level of strength or an inability to complete a physical task that was previously achievable [5]. In terms of football, fatigue can appear because some players can be unable to assimilate matches and training loads, increasing the risk of injury and underperformance [6]. In this sense, coaches and researchers agree about the importance of managing fatigue to achieve optimal muscular adaptations, increase progress, prevent overtraining, decrease injury risk and improve performance [7]. However, very few studies have actively investigated how to measure fatigue and adjust training accordingly. 

Fatigue can be measured through internal and external loads [5]. In football, there are several internal load methods that are very popular to measure fatigue and response to exercise; however, most of them present some disadvantages, so they should be combined with other methods. Heart rate measurements are the most common, but they are imprecise in high-intensity actions and sprints, which are crucial aspects of football [8]. Blood and bone markers are more precise, but they are difficult to extract on a weekly basis (price and time), and some players can feel uncomfortable being exposed continuously to them. Therefore, extracting them whenever the physical status of the player needs to be known can be unsustainable during regular-season training [9]. Questionnaires are very simple to administer but particularly subjective, introducing a high risk of bias [10]. 

Conversely, there are some physical tests that can help to check the current physical condition of the athletes through external loads. It has been evidenced that velocity loss is a reliable marker of neuromuscular fatigue, which is described as a decrease in the physical and mental functions [11]. This is why most of the scientific literature has used the repeated sprint ability (RSA) test to measure fatigue, especially in team sports such as football; this is because RSA and the ability to exercise at high intensity are key capacities for optimal performance, and a decreased sprint repetition capacity is a good indicator of fatigue in sports [12]. However, this method can lack precision in some situations, as the scientific literature has found that sprint performance can be maintained in situations of neuromuscular fatigue after match play [13]. This is why some coaches incorporate a stretching–shortening movement, such as a countermovement jump (CMJ) to detect fatigue, as it can highlight impairments in jump performance between two events [14]; a good relationship between sprint ability and CMJ capacity has been verified [14].

Therefore, scientific evidence has shown CMJ performance to be an objective marker of fatigue and supercompensation, as neuromuscular fatigue has been associated with a decrease in the average CMJ height [15], causing this method to be used frequently to assess neuromuscular fatigue [16]. However, when using RSA to assess neuromuscular fatigue, the number of sprints induces great variability between athletes [17]. In addition, when using CMJs, it is important to consider that the same fatiguing stimuli can elicit different effects between individuals, and sometimes athletes can adjust their jumping strategy to maintain their performance [18]. Moreover, players’ need to exert maximal effort to measure fatigue is not optimal, as this effort increases injury risk. Hence, the use of other technologies which can provide information and help coaches without the need to perform a physical test, can be considered. In this sense, one option is infrared thermography (IRT), which is a non-radiating, contact-free, safe and non-invasive technology that monitors physiological variables through the control of the skin temperature [19].

This technology gauges the correlation between muscle activation and skin temperature, as muscle and skin temperature are directly correlated. The information this technology provided is based on the hyperthermic and hypothermic responses of the skin [20], due to the fact that thermography is a reliable method to assess skin temperature [21]. Moreover, athletes are presumed to keep their thermal pattern constant in baseline conditions, and thermal asymmetries in the bilateral body areas (e.g., ankle, knee, hamstring or elbow) are linked to factors related to injuries, such as inflammation or secondary trauma, as in normal conditions, the temperature of both sides of the bilateral areas should increase equally [22]. IRT can detect these asymmetries by comparing bilateral body areas, showing potential injury risk due to incorrect work assimilations provoked by factors such as excessive training, poor technique or muscle overload [23]. 

The relevance of IRT is that it can detect temperature asymmetries (and consequent risks) before other markers such as pain, making this method extraordinarily effective and applicable in preventing injuries [23]. If IRT provides coaches with the ability to detect impairments in bilateral body areas before pain, it can allow them to modify the training load proactively, decreasing injury risk and increasing performance [15]. This advantage is especially remarkable in sports like football, in which high-intensity interval training combined with weekly competition can lead the locomotor system to its anatomical and physiological limit [19], exponentially increasing the risk of minor injuries, overuse injuries, lower-limb injuries and muscle strains [19]. In recent years, authors have explained the efficiency of this technology for injury prevention in medicine [24]; however, IRT has not been widely investigated in athletes such as football players, as only a couple of studies have explored the use of IRT for preventing football injuries [25,26]. Nevertheless, none of the studies performed with thermography have aimed to compare thermography results with the information that external loads provide about players’ physical condition in order to adapt their training loads in accordance with the results; therefore, this concept demands research. 

In this sense, the aim of the study was to determine if there is a relationship between thermal asymmetries provided by thermography and performance in a repeated sprint ability (RSA) test and in countermovement jumps (CMJs) before and after the RSA test. If thermography can predict these tests´ performance without the need to execute them, it can offer coaches the possibility of adapting players’ training loads without the need for them to exert maximal efforts. 

## 2. Materials and Methods

### 2.1. Experimental Approach to the Problem

**Figure 1:**
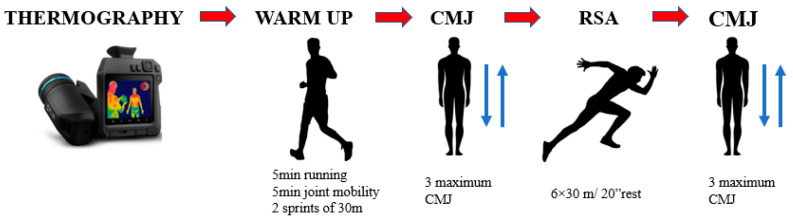
Flowchart of the protocol.

This study used repeated measures within participants to determine the relationship between skin temperature and/or thermal asymmetries in the bilateral body areas, CMJ performance and RSA performance. The protocol consisted of a thermography session of the lower limbs (anterior and posterior parts) of each player in basal conditions after 48 h resting, followed by a standardized warm-up that included 5 min of continuous running, 5 min of joint mobility and two sprints of 30 m with a recovery process of 2 min, and a vertical jump test (CMJ) + repeated sprint ability (RSA) test (6 × 30 m/20″) + vertical jump test (CMJ) (See Figure 1 for more clarity). 

### 2.2. Participants 

The participants were 20 male football players from a professional team of the Smartbank league (the second Spanish football division) (age 28.9 ± 3.9 years; height 178.7 ± 9 cm; body mass 74.8 ± 6.4 kg). Players with recent injuries or pain were excluded, as they could interfere with the results. Each player signed informed consent with an explanation of the study procedure as well as the associated risks. 

### 2.3. Ethical Statement

The study was conducted according to the requirements of the Declaration of Helsinki (2013) and was approved and followed the guidelines stated by the Ethics Committee of the European University of Madrid (CIPI35/2019). The research also received formal approval from the professional football club involved.

### 2.4. Measures 

#### 2.4.1. Thermography 

**Figure 2:**
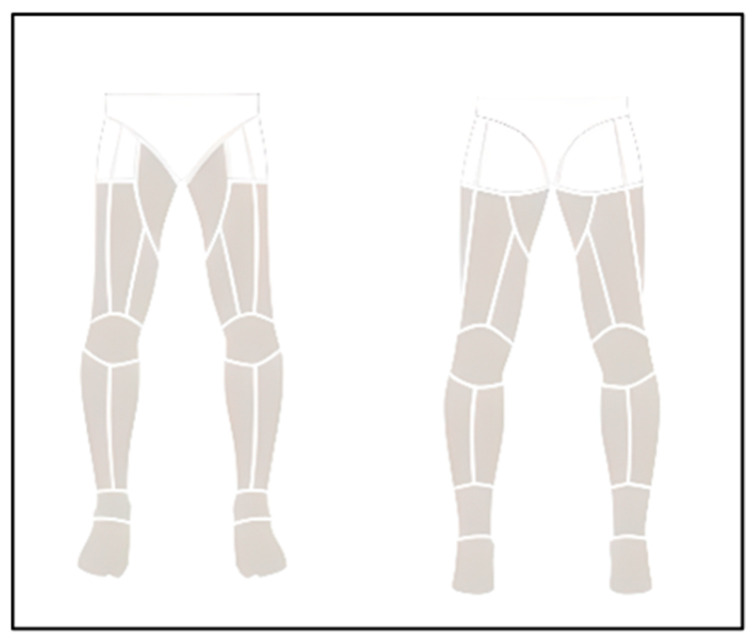
Measurement areas provided by the Thermohuman software (anterior and posterior parts).

The collection of thermographic data followed the standards proposed by the consensus statement of TISEM on the measurement of human skin temperature [27]. Thermograms were evaluated before the protocol and used as a control variable prior to testing. Thermograms were performed in an air-conditioned room; the temperature was set at 22 °C (±1.5 °C) with about 40–60% of relative humidity, and the skin temperature of the lower limbs at the anterior and posterior parts was recorded. The thermal camera FLIR T420bx (FLIR Systems, Sweden) with a resolution of 320 × 240 pixels was placed 3 m away from the participants and at a perpendicular angle to them. The players were instructed to rest 24 h prior to the thermograms and to avoid behaviours that could interfere with the assessment of thermal images, such as drinking alcohol, smoking or consuming caffeine. During testing, the participants were dressed in underwear and were barefoot, so selected areas of skin were continuously exposed during the exercises and measurements. Following the Thermohuman technology protocol, to facilitate the analysis, the body regions of interest (ROIs) analysed included the thighs, calves and hamstrings, as these are the main muscles of the lower limbs [23]. Computerized image analysis allowed the selection of the measurement area in the thermograms (see Figure 2). The areas were analyzed with the Thermohuman software (PEMA THERMO GROUP, Spain). From the analyzed ROIs, average, minimum, maximal temperature was extracted to calculate thermal asymmetries between bilateral ROIs. 

The players were gathered into pairs in subgroups (low [<0.2 °C] vs. high thermal asymmetry [≥0.2 °C]) based on the results of the thermography session. Previous literature has considered clinically significant skin temperature asymmetry to be over 0.5 °C, but it was decided to establish the cut-off point at 0.2 °C, because the sample is from a professional club and the members were highly supervised. The physical department of the club made the political decision to start following, taking care of and monitoring players when they have ≥0.2 °C asymmetry. Thus, not many players reached 0.5 °C asymmetry. Therefore, the sample was divided into low and high thermal asymmetry of thighs, hamstrings and calves. The players were grouped in terms of their asymmetry for each muscle group; accordingly, one player could be in the high-asymmetry group for one of the studied muscles and the low-asymmetry group for another muscle. 

#### 2.4.2. Vertical Jumps

Players completed 2 CMJ tests: (1) after warming up and (2) after the RSA test, with some minutes to recover from it. To measure the height of the jumps, infrared technology was used (Optojump Next, Microgate, Bolzano, Italy). The participants were already familiar with the movement, and they were instructed to undertake it in the most precise way, keeping their hands on their hips to eliminate the influence of arm movement on the jump performance. Each player performed three jumps before and after the RSA test (with 2 min of recovery between jumps). The average of the three jumps was calculated for the statistical analysis.

#### 2.4.3. Repeated Sprint Ability (RSA)

The RSA test included six sprints of 30 m with 20 s recovery between sprints. Two pairs of photocells (Witty, Microgate, Bolzano, Italy), placed at 0 and 30 m, were used. The following measures were calculated: sprint time (RSAt), best sprint time, average time, total time, percentage difference between the first and the rest of the sprints during the RSA test − %Diff − [((sprint time − first sprint time)/first sprint time) × 100] and percentage difference between the best and the rest of the sprints during the RSA test − %Best − [((sprint time − best time)/best time) × 100]. The previous two sprints performed during the warm-up were used as a control measure to ensure that players performed the RSA test at maximum speed. If the time of the first RSA test sprint was longer (>5%) than the best individual sprint performed before the start of the test, the RSA test was not considered valid, and the player had to repeat the test after 5 min of recovery. 

### 2.5. Statistical Analysis

The data are presented as means ± standard deviations. The normal distribution of the variables was confirmed by histogram charts and the Shapiro–Wilk distribution test. A mixed two-way analysis of variance was performed to analyse the effect of the level of thermal asymmetry on the RSA variables depending on the sprint number. The thermal asymmetry group was used as an independent factor, and the sprint number as a repeated measured factor to control the interaction of the thermal asymmetry group with the sprint progression. Bonferroni post hoc pairwise comparisons were undertaken to compare the RSA variables of the two groups of thermal asymmetries in each of the sprints, including the confidence interval (95%). The effect size (ES) was calculated for all the inference tests using the partial eta squared (ηp2 = ES) value with the following interpretation: small (ES = 0.01–0.059); medium (ES = 0.06–0.14); and large effects (ES > 0.14). The level of significance was set at *p* < 0.05 for all the tests, and all the data were statistically analysed using SPSS V24.0. (IBM Corp. Released 2016. IBM SPSS Statistics for Windows, Version 24.0. Armonk, NY: IBM Corp.) 

## 3. Results

**Figure 3:**
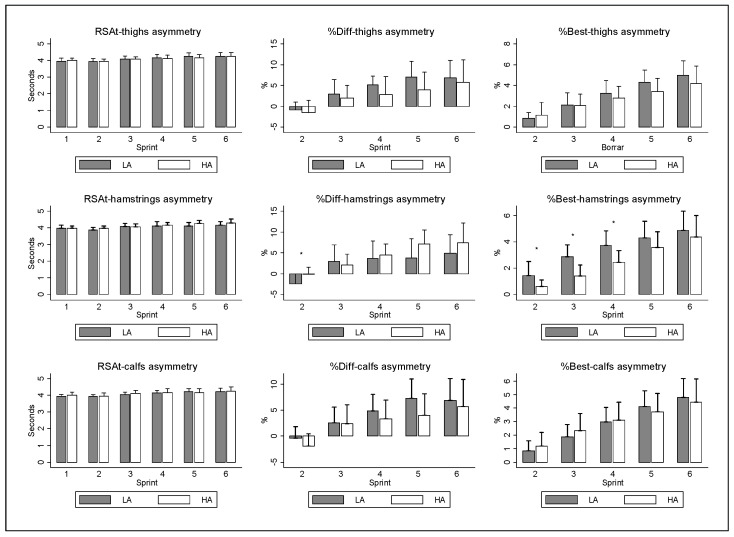
Time differences based on low (LA) and high (HA) thermal asymmetry groups. * Significant differences between groups (*p* < 0.05). (RSAt = sprint time). (%Diff = [((sprint time − first sprint time)/first sprint time) × 100]). (%Best = [((sprint time − best time)/best time) × 100].

The normal distribution of the variables was confirmed by histogram charts and the Shapiro–Wilk distribution test (the statistic varies between 0.926 and 0.984, *p* > 0.005 in all cases). Figure 3 shows the results for the RSA variables: the sprint time (RSAt), the percentage difference between the first and the rest of the sprints during the RSA test − %Diff − [((sprint time − first sprint time)/first sprint time) × 100] and the percentage difference between the best and the rest of the sprints during the RSA test − %Best − [((sprint time − best time)/best time) × 100]. The results are calculated based on thermal asymmetry clusters (a group with low asymmetry and a group with high asymmetry in each of the three muscle groups). Thigh and calf asymmetry did not show a significant effect on any variable (*p* > 0.05). Moreover, no significant interaction was found between thigh and calf asymmetry and the number of sprints (*p* > 0.05). Conversely, hamstring asymmetry did not show a significant effect in the RSA test and %Diff (*p* > 0.05), but it did in %Best (F = 6.59; *p* = 0.018; ES = 0.25), highlighting fewer differences in high asymmetry with respect to the best sprint. Furthermore, a significant interaction was found between hamstring asymmetry and sprint number 2 in the RSAt (F = 2.42; *p* = 0.041; ES = 0.11) and in %Diff (F = 2.50; *p* = 0.049; ES = 0.11) but not in %Best (*p* > 0.05). Regarding the pair-wise comparison, the hamstring high-asymmetry group showed a higher %Diff in sprint 2 (F = 7.40; *p* = 0.013; IC: −4.20 to −0.55; ES = 0.27), and there was also a higher %Best in the hamstring low-asymmetry group in sprints 2 (F = 5.76; *p* = 0.026; IC: 0.11 to 1.55; ES = 0.22), 3 (F = 15.59; *p* = 0.001; IC: −1.55 to −0.11; ES = 0.44) and 4 (F = 9.36; *p* = 0.006; IC: 0.67 to 2.22; ES = 0.32).

Table 1 shows the differences in CMJ variables between all the defined groups. There are no significant differences in any of the variables between the low and the high thermal asymmetry group for thighs and calves. However, for hamstrings, while there are no significant differences between the low and the high thermal asymmetry group in the CMJ Pre-RSA and Diff CMJ tests (*p* > 0.05), the low asymmetry group has significantly higher values for the CMJ Post-RSA (F = 7.55; *p* = 0.013; IC: 1.06 to 7.84; ES = 0.28).

## 4. Discussion

The purpose of this study was to determine if there is a relationship between thermal asymmetries provided by thermography and performance in a repeated sprint ability (RSA) test and in countermovement jumps (CMJs) before and after the RSA test. The results of the RSA test showed a significant interaction between hamstring asymmetry and the number of sprints in the RSAt and in the % Diff. The hamstring high-asymmetry group showed a higher %Diff in sprint 2, which means that there is a significative longer time with respect to sprint 1 (worse performance) than in the low-asymmetry group. On the other hand, the hamstring low-asymmetry group reported a higher %Best in sprints 2, 3 and 4. The cause of this difference in the hamstring %Best may be attributable to the fact that the low-asymmetry group had the best sprint, which was very fast; therefore, the following best ones have a significant difference (worse). On the other hand, no differences were found in the thigh and calf groups for any variable. The reason why the only thermal asymmetry that influenced the performance was that of hamstrings could be their importance in sprinting, as they play a crucial role in generating force in the propulsive part of the sprint [28] and are clearly the most frequently injured muscle during sprinting [4]. The majority of hamstring muscle injuries occur while the athlete is running at maximal or close to maximal speeds [29] Many investigations have measured hamstring activity during sprinting with electromyographic (EMG), and found that the hamstrings are active from the middle of the swing to the final stance Some of these studies have reported that peak activity occurs during terminal swing [30], whereas others have found it to occur during stance [31]. Proof of the effort that these muscles exert in the sprint is that an increase in running speed of 80–100% is linked with an increase in net hamstring muscle force and energy absorption of 1.4 and 1.9 times, respectively [32]. However, this study took data from athletes running on a treadmill, and the mechanical properties of treadmill surfaces are different from those of the surfaces on which the athletes usually train and compete (e.g., artificial turf or an athletic track) [33]. This factor may influence running anatomy, so the results should be interpreted with caution. 

Regarding the CMJ results, the low thermal asymmetry hamstring group has significantly higher values in the CMJ post-RSA, while in the calf and thigh groups, there are no significant differences in either CMJ pre- or CMJ post-RSA. Thus, the high thermal asymmetry hamstring group’s performance was similar to that of the low thermal asymmetry hamstring group before the RSA, and then its performance decreased in the CMJ post-RSA. This can be indicative of fatigue, as a decreased average CMJ jump height can indicate neuromuscular fatigue [34]. The reason why significant differences are only found in the high-asymmetry hamstring group (and not in the calf and thigh groups) may be that hamstrings are the most implicated and frequently injured group in sprinting [28], while calves and thighs seem to play a less crucial role in sprinting; therefore, asymmetry takes longer to turn into fatigue when performing an RSA test, as they are less implicated in this activity. 

Although the results of this study seem to provide evidence in terms of using and interpreting thermography to assess RSA and CMJ performance in elite football players, the results must be interpreted with caution, as certain limitations should be considered. First, thermography was carried out in just one session; applying thermography in a higher number of sessions would allow us to corroborate the results of the study. Second, a low asymmetry cut point was established, and settling a higher asymmetry cut point would probably show stronger correlations. Finally, the sample was very small, so future research should be carried out to look for evidence and to check whether these results apply to different genders, ages and competitive levels.

From a practical point of view, if football coaches can access the thermographs of their players, they should focus especially on hamstrings (rather than calves and thighs) and modify the training if necessary. These muscles appear to be the ones with more influence on the test results, as they are the only ones for which thermal asymmetry of over 0.2 °C had an effect. 

## 5. Conclusions

Thermography can provide information about performance in CMJ and RSA tests for hamstring asymmetries over 0.2 °C; meanwhile, calf and thigh asymmetries over 0.2 °C do not seem to have a relationship with performance in these tests. In short, thermography results have some correlation with CMJ and RSA performance, but stablishing higher cut off points would probably allow us to find stronger correlations for hamstring asymmetries, and maybe some correlation for calf and thigh asymmetries which would be helpful for coaches and athletes. 

## Figures and Tables

**Table 1:** Differences in CMJ variables depending on low and high asymmetry.

		Low Asymmetry	High Asymmetry
Thigh asymmetry	CMJ Pre	39.30 ± 3.59	38.78 ± 4.74
	CMJ Post	34.35 ± 4.07	35.13 ± 4.64
	Diff CMJ	−4.96 ± 3.12	−3.65 ± 3.17
Hamstring asymmetry	CMJ Pre	40.80 ± 3.62	37.79 ± 3.95
	CMJ Post	37.23 ± 3.78	32.78 ± 3.59 *
	Diff CMJ	−3.58 ± 3.28	−5.01 ± 3.01
Calf asymmetry	CMJ Pre	38.98 ± 4.41	39.19 ± 3.77
	CMJ Post	34.15 ± 3.97	35.27 ± 4.64
	Diff CMJ	−4.83 ± 2.53	−3.92 ± 3.77

* Significant differences between groups (*p* < 0.05).

## Data Availability

The data presented in this study is available on request from the corresponding author.
